# Lessons Learned From the Creation of a Business of Medicine Course

**DOI:** 10.15694/mep.2019.000154.1

**Published:** 2019-07-18

**Authors:** Joshua Daily, Esther Teo, Carol Thrush, Jason Mizell

**Affiliations:** 1 University of Arkansas for Medical Sciences

**Keywords:** business of medicine, personal finance

## Abstract

This article was migrated. The article was marked as recommended.

Knowledge, attitudes, and skills required to successfully manage business and personal finances are rarely taught within traditional medical education. This has contributed to low financial literacy, high stress related to educational debt, and burnout among physicians. To address this deficiency, we created the Business of Medicine course for fourth-year medical students which teaches basic business and personal finance topics. As we have reflected on lessons learned in the creation and implementation of this course, we have recorded them for the benefit of others who desire to partner with us in teaching this important topic to the next generation of physicians.

## Introduction

As a new practicing surgeon, I (JM) came to the realization that in spite of being competent and highly trained in medicine, I knew almost nothing about the business side of medicine or physician personal finance. I had completed four years of college, four years of medical school, and six years of residency and fellowship, and had never been taught how to bill and code for services I provided, manage a practice, budget, save, invest, or handle a large income and the expectations of a physician lifestyle. Unfortunately, few of my colleagues and mentors seemed financially astute either. I, therefore, embarked on a journey of financial self-study that was quite difficult as I had neither a guide nor many physician-specific financial resources. This deficiency coupled with my role as an academic surgeon inspired me to create an introductory, physician-specific, financial education curriculum for our surgery residents ( Mizell *et al.*, 2014). As I learned more about our residents’ financial status, it became clear to me they were making major financial mistakes that potentially could have been prevented with education on basic concepts during medical school. So with support from my institution, I used the resident course as a template to create a fourth-year medical student elective titled “The Business of Medicine (BoM).”

In the first year of the BoM course, 66 of the approximately 160 fourth-year medical students at the University of Arkansas for Medical Sciences completed the course. In subsequent years, student attendance grew to 93, 125, and most recently 127 participants. Our group has previously published a description of the course curriculum and the initial program evaluation results ( Mizell, Thrush and Steelman, forthcoming 2019). We continuously update the course’s structure and content in response to feedback from the participants, survey data, and our own failures. As we have reflected on lessons learned during the creation and implementation of the course, we have recorded them for the benefit of those who desire to partner with us in teaching this important topic to the next generation of physicians.

## 1. Physicians and Medical Trainees Need and Want Basic Personal Financial Education

Early on, it became clear there was both strong demand for the information we were teaching and a significant knowledge gap among course participants. This knowledge gap was most clearly seen in our pre-course surveys, where only 12% of students reported “somewhat” or “strongly agree” that they felt competent managing their finances. Course enrollment has grown from 41% to 79% of the fourth-year class, making it the most popular elective at our institution. In addition, many students, residents, fellows, and practicing physicians voluntarily audit the course. While initially the curriculum was split evenly between business and personal finance, we have increased the number of lectures focused on basic personal finance. Participants routinely request more exposure to topics such as budgeting, saving, debt management, and investing. Now the distribution is approximately 60% personal finance and 40% business-related topics.

## 2. Medical Students Are Highly Concerned About Their Debt, and They Should Be

Physician educational debt is rising at a rate that far outpaces national inflation. In 2018, 75% of graduating United States medical students had educational debt with a median debt burden of $200,000 ( Rowe and Wisniewski, 2018). The impact of this is substantial as high levels of physician debt are associated with higher rates of burnout, lower quality of life, and lower satisfaction with work-life balance ( West, Shanafelt and Kolars, 2011). This reality is not lost on medical students. The vast majority of the BoM course participants have educational debt and report this is a significant source of stress. These concerns extend past graduation. In a recent survey of our former BoM students, the majority (64%) of free text comments regarding their greatest financial concerns were specifically about issues related to student debt. We have previously created and published a proposed strategy for student debt management ( Lynch *et al.*, 2018), but this conundrum is far from solved. While physician debt has obvious public policy considerations and varies by country, residents and medical students with debt need dedicated education on this important topic.

## 3. Expectations Regarding the Physician Lifestyle Start Early

We are frequently amazed by the consumption lifestyle of students, trainees and new attending physicians as evidenced by their luxury automobiles, expensive vacations, $5 cups of coffee, $1,000 phones, and large homes. In the vast majority of situations, they are either financing these expenditures with debt or forgoing paying off debt to make these purchases. They do not seem to recognize that due to their significant negative net worth, they are poorer than the homeless man they passed on the street. The prevailing belief seems to be that they will be or are rich doctors, and thus they choose a consumption lifestyle commensurate with this expectation. However, what is usually missed is that due to the significant delay in both earning and saving, the impact of student debt, and the reality of reduced physician income among primary care and many subspecialties, even after residency most physicians cannot choose an upper-class consumption lifestyle while still adequately saving for retirement and paying off debt ( Daily, Gutierrez and Bolin, 2018). What should be even more obvious is that very few medical trainees can afford upper-class lifestyles. In the BoM course, we have addressed this expectation with scenarios showing actual physician income and expense data, research demonstrating a threshold beyond which consumption does not increase happiness ( Kahneman and Deaton, 2010), and a strategy of slowly growing into a new attending salary while rapidly reducing debt. While these educational methods have been helpful for our students, we believe this is an area in need of further focused education and research.

## 4. Sources of Trustworthy Physician Financial Education Are Rare

In arranging speakers for the BoM course, we have been surprised by both the frequent requests from financial salesmen to speak to potential prospective physician clients and the difficulty in identifying well-trained experts on physician financial issues. The neurologist and financial author William Bernstein put it this way, “If you act on the assumption that every broker, insurance salesman, mutual fund salesperson, and financial advisor you encounter is a hardened criminal, you will do just fine” ( Bernstein, 2009). This highlights the difficulty in identifying trust-worthy, well-trained educators for the BoM course. We have ended up relying on physicians with expertise in business and finances and have utilized a few select finance professionals after significant vetting by the course director. In addition, *The White Coat Investor* ( Dahle, 2014), which is written by an emergency medicine physician and financial blogger, has served as the primary text for our course.

## 5. A Single Course on Finances Is Helpful but Inadequate

Unfortunately, financial material is rarely taught in pre-medical classes, medical school, residency, fellowship, or continuing medical education courses. While the BoM course attempts to correct this deficit and introduce financial topics, 20 hours is insufficient to provide thorough, comprehensive financial education for all stages of a physician career. Because of time and logistical limitations, the BoM course is taught in a lecture format, which may limit knowledge acquisition and retention. In addition, the financial needs and interests of 4 ^th^-year medical students are different from those of attending physicians. While the initial goal of the BoM course was to provide comprehensive financial education, we have since adjusted the objectives to teach basic financial knowledge and skills and to introduce resources for further financial education. In an attempt to provide more comprehensive and longitudinal financial education, we have also recently created a medical school Honors in Finance track. This is available for students as early as their first year of medical school and consists of four years of quarterly didactic sessions, reading assignments, and mentor meetings. The ultimate goal is for participants to complete a personal financial plan prior to graduation. Despite these aforementioned limitations, we have still observed clear knowledge acquisition and behavioral change among our BoM students. In a recent post-course survey, 95% of students reported making at least one behavioral change as a result of the course. However survey comments like the following were very common: “Taking the BoM course was the catalyst that fostered my interest in personal finance.” It is clear that ongoing financial education is necessary for all physicians. Our hope is that our BoM course and others like it at all levels of medical training may address the significant need.

## Conclusion

The BoM course has successfully led to behavioral changes and reduced stress among course participants ( Mizell, Thrush and Steelman, forthcoming 2019). However, the work is far from complete as the course’s long-term outcomes remain unknown, and few other courses providing physician-specific financial education exist. Our hope is that other educators and researchers will partner with us in teaching and studying this important topic so that future generations of physicians may be equipped to manage this important aspect of their lives and careers.

## Take Home Messages

• Physicians and Medical Trainees Need and Want Basic Personal Financial Education
• Medical Students Are Highly Concerned About Their Debt, and They Should Be
• Expectations Regarding the Physician Lifestyle Start Early
• Sources of Trustworthy Physician Financial Education Are Rare
• A Single Course on Finances Is Helpful but Inadequate

## Notes On Contributors

Joshua Daily, MD, MEd is an Assistant Professor of Pediatrics and Pediatric Cardiologist at the University of Arkansas for Medical Sciences and Arkansas Children’s Hospital ( https://orcid.org/0000-0002-0159-0247).

Esther Teo, MD is an Assistant Professor of Surgery and Burn Surgeon at the University of Arkansas for Medical Sciences and Arkansas Children’s Hospital.

Carol Thrush, EdD is an Associate Professor and Medical Educator at the University of Arkansas for Medical Sciences.

Jason Mizell, MD is an Associate Professor of Surgery, Colon and Rectal Surgeon, and the Business of Medicine Course Director at the University of Arkansas for Medical Sciences.

## References

[ref1] Bernstein W. J. (2009) The investor’s manifesto: preparing for prosperity, Armageddon, and everything in between. John Wiley & Sons.

[ref2] Dahle J. M. (2014) The White Coat Investor: A Doctor’s Guide to Personal Finance and Investing. Createspace Independent Pub.

[ref3] Daily J. Gutierrez S. C. and Bolin E. (2018) The Most Important Financial Year of Your Life: Recommendations for the Transition From Resident to Practicing Pediatrician. Clinical Pediatrics. 0(0),57(11),1266–1268. 10.1177/0009922818772055 29695167

[ref4] Kahneman D. and Deaton A. (2010) High income improves evaluation of life but not emotional well-being. Proceedings of the National Academy of Sciences. 107(38), pp.16489–16493. 10.1073/pnas.1011492107 PMC2944762 20823223

[ref5] Lynch A. Best T. Gutierrez S. C. and Daily J. A. (2018) What Should I Do With My Student Loans? A Proposed Strategy for Educational Debt Management. Journal of Graduate Medical Education. 10(1), pp.11–15. 10.4300/jgme-d-17-00279.1 PMC5821025 29467967

[ref6] Mizell J. Thrush C. and Steelman S. ( forthcoming 2019) The business of medicine - A course to address the deficit in financial knowledge of fourth year medical students. Journal of Medical Practice Management.

[ref7] Mizell J. S. Berry K. S. Kimbrough M. K. Bentley F. R. (2014) Money matters: a resident curriculum for financial management. Journal of Surgical Research. 192(2), pp.348–355. 10.1016/j.jss.2014.06.004 25005821

[ref8] Rowe S. and Wisniewski S. (2018) AAMC data book: Medical schools and teaching hospitals by the numbers. Association of American Medical Colleges.

[ref9] West C. P. Shanafelt T. D. and Kolars J. C. (2011) Quality of Life, Burnout, Educational Debt, and Medical Knowledge Among Internal Medicine Residents. Journal of the American Medical Association. 306(9), pp.952–960. 10.1001/jama.2011.1247 21900135

