# Supplementary material for: Lessons Learned From the Creation of a Business of Medicine Course
Source: MedEdPublish (2016). 2019 Jul 18;8:154. [Version 1] doi: 10.15694/mep.2019.000154.1 (PMC10712494; doi:10.15694/mep.2019.000154.1)
Supplement: Peer review history

# Reviewer report: Reviewer response for version 1

**DOI:** 10.21956/mep.19884.r30081

**Published:** 2019-08-09

**Authors:** Paolo Colet

**Affiliations:** 1 Nazarbayev University

This review has been migrated. The reviewer awarded 5 stars out of 5 The article was brief but comprehensive in sharing valuable and tested business course for the physicians. Also, the outcomes of the course mentioned were discussed in line with the available literature. Interestingly, the strategy of imparting financial education to physicians seems to be successful and beneficial to learners. Indeed, such a course can be useful to learners from other health-related programs as well.

Reviewer Expertise:

NA

No decision status is available

# Reviewer report: Reviewer response for version 1

**DOI:** 10.21956/mep.19884.r30080

**Published:** 2019-07-30

**Authors:** Jayne Garner

**Affiliations:** 1 university of liverpool

This review has been migrated. The reviewer awarded 4 stars out of 5 Thank you for a very relevant and insightful paper, raising a number of issues for medical educators and universities. While the author experience is based in the US, I feel a number of points are transferable internationally. In my experience, medical students and recent graduates can struggle with their personal finances, especially if they are from diverse backgrounds and cannot depend upon family to help them. From a business perspective, clear advice about contracting processes and managing budgets is also rare in undergraduate medical education. Your paper is well written and evidenced - thank you.

Reviewer Expertise:

NA

No decision status is available
